# Population-based Seroprevalence of HTLV-I Infection in Golestan Province, South East of Caspian Sea, Iran

**Published:** 2013-03

**Authors:** Khodaberdi Kalavi, Abdolvahab Moradi, Alijan Tabarraei

**Affiliations:** 1 Infectious Diseases Research Centre, Golestan University of Medical Sciences, Gorgan, Iran

**Keywords:** HTLV-I, Seroprevalence, ELISA, Western Blot, Golestan, Iran

## Abstract

***Objective(s):*** Human T-cell lymphotropic virus type-1 is an oncornavirus that causes adult T cell leukemia (ATL) HTLV-I-associated myelopathy⁄tropical spastic paraparesis (HAM/TSP). Golestan province is located in North West of Khorasan province known as an endemic area for HTLV-I in Iran. This study aimed to evaluate seroprevalence of HTLV-I in Golestan province.

***Materials and Methods:*** In this cross-sectional descriptive study in 2007, blood samples were collected from 2034 healthy people residing in different parts of Golestan province. Sera were assessed for HTLV-I/II–specific antibodies by ELISA method and reactive samples were confirmed by Western blot. Demographic and serologic data were entered in SPSS version 11.5 and statistical analysis was performed.

***Results:*** An overall HTLV-I/II prevalence of 0.7% was observed in 15 cases by ELISA. Six out of 15 were confirmed as HTLV-I by western blot. Regional variation in the prevalence of HTLV-I was observed; 0%, 0%, 0.1%, 1.9%, 0.3%, 0%, and 2.6% tested HTLV-I-positive from west to east of Golestan Province regions, respectively. Seropositivity increased with age. No association between HTLV-I infection and sex status was detected.

***Conclusion:*** Highest rate of HTLV-I seroprevalence was shown in east of this region located in neighborhood with Khorasan province, the only confirmed endemic area in Iran. It seems that eastern area of our province is endemic for HTLV-I. Further comprehensive detailed epidemiological and molecular studies are recommended.

## Introduction

Human T-cell lymphotropic virus type-1 (HTLV-I) is a member of Retroviridae family which has been discovered as the first human retrovirus. This oncornavirus causes the T cell malignancy associated with two main diseases; adult T cell leukemia (ATL) and HTLV-I-associated myelopathy/tropical spastic paraparesis (HAM/TSP) (1, 2). Worldwide estimation of HTLV-I infected people is approximately 20 million and it has been suggested more than 90% of them remain asymptomatic carriers during their lives. Geographic distribution of the virus indicates that southwestern Japan, parts of Africa, the Caribbean islands, and Central and South America are the main endemic regions of HTLV-I in the world (3, 4). However, the data should be interpreted based on the population selection criteria and the differences in the diagnostic strategies. Mainly, the data provided from the serological screening of healthy blood donors is the basis for the estimation of the global prevalence of HTLV-I, which tends to underestimate the prevalence of the virus in the population (5, 6). HTLV-I is transmissible through breast milk, semen, and HTLV-I carrier’s lymphocytes and all of transmission routes efficiently localize HTLV-I infection foci within particular family ⁄ethnic groups (7-9). On the other hand, it is considered to study the global prevalence of HTLV-I infection in the context of ethnicity-based, as a new paradigm for cancer research for host factor interaction assay with exogenous carcinogens (10).

Iran has been introduced as an endemic area based on studies reported from Mashhad in Khorasan (A province of Iran recently divided into three provinces) located in the northeast of Iran (11, 12). The latest report from Mashhad showed the overall prevalence of 2.12% HTLV-I infection in the whole population (13). The previous HTLV-I infection prevalence report from Golestan has been limited to the Thalassemia patients with 4.4% (14). Golestan is another province of Iran located in the southeast of Caspian Sea next to Khorasan. Different ethnic groups are living in this province and emigration from the east and northeast of the country to this region is common. In the endemic developed countries and some developing countries, HTLV-I screening of blood donors was already performed. The province of Golestan has not performed HTLV-I screening of blood donors yet. This study aims to evaluate the population-based HTLV-I seroprevalence in the province of Golestan.

## Materials and Methods

From all of the seven main cities with an estimated population of 1.5 million, 2034 individuals were selected through multistage cluster sampling in 2007. Demographic information, such as sex, age, and residency status, was collected. The study was approved by Deputy of Research of Golestan University of Medical Sciences regarding scientific and ethical issues. Informed consent was obtained from all participants. Five ml of blood samples were obtained from each individual. Serum was separated through centrifugation and was stored at −20^◦^C. Serum samples were screened for the presence of anti-HTLV-I antibodies with the HTLV I/II enzyme linked immunosorbent assay (ELISA) (DIA.PRO, Diagnostic Bio probes Srl, Italy) according to the manufacturer’s instructions. All reactive samples on serologic screening were tested further through Western blot (WB) analysis according to the manufacturer’s instructions (HTLV BLOT 2.4, Gene labs Diagnostics). Descriptive data was summarized as the mean, standard deviation, and/or percentages were analyzed by SPSS 11.5 using Chi-square and t-tests. A *P-*Value of <0.05 was considered statistically significant.

## Results

Out of 2034 people studied in this population, based seroepidemiology, 848 cases were male (41.7%) and 1186 participants were females (58.3%) with the mean age of 38.66±16.54 years. All serum samples were analyzed for anti-HTLV antibodies.

In the primary screening of the samples by ELISA, 15 (0.7%) were positive for HTLV1/2 antibodies. The Western blot results confirmation demonstrated that 6 (0.3%) out of 15 ELISA positive specimens were HTLV-1 positive, but HTLV positivity was not confirmed in 9 cases (0.4%). According to the Western blot results, the overall prevalence of the HTLV-I infection in the population under study was 0.3% (6/2034). We did not find an indeterminate result by Western blot. The HTLV-I infection rate for females was 0.3% (3/1186) and for males was 0.4% (3/848). No significant difference in the seroprevalence was observed between males and females (Table 1).

**Figure1:**
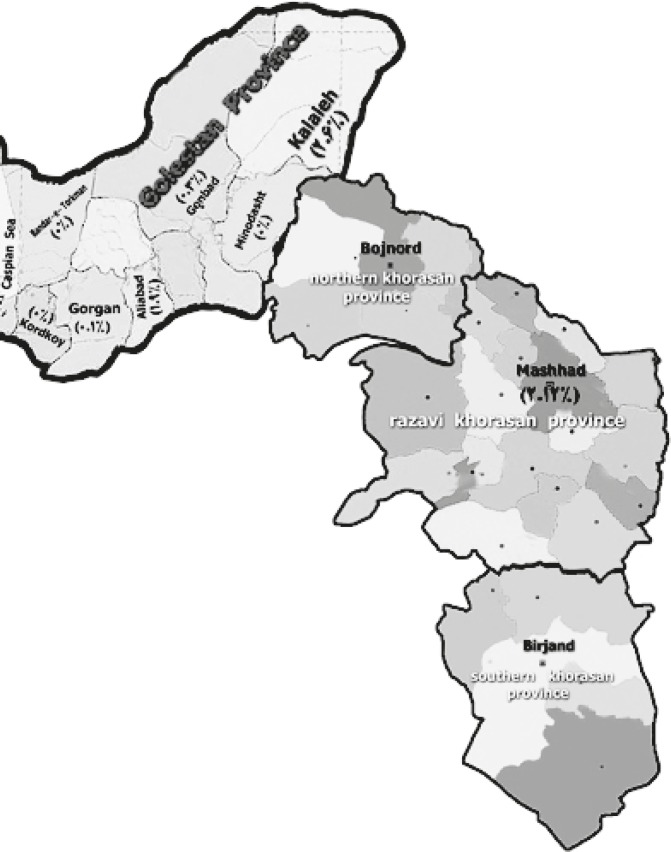
Map of HTLV-I Infection distribution in Golestan and Khorasan provinces, Iran

Regional variation in the prevalence of HTLV-I was observed to be 0%, 0% 0.1%, 1.9%, 0.3%, and 2.6% tested HTLV-I-positive from west to east of Golestan regions, respectively (Figure 1). Seroprevalence of the disease increased with age, as observed more among those older than 20 years old(Table 1).

**Table 1:** Demographic factors related to HTLV-I Infection in the general population of Golestan province, Iran

Variable	No.	Positive (%)	*P*-Value
Sex			
Male	848	0.4	
Female	1186	0.3	0.49
Age (years)			
1-10	44	0	
11-20	243	0	
21-30	506	0.4	
>30	1237	0.4	0.25
Residency area			
Bandar Turkman	298	0	
Kord-Koy	50	0	
Gorgan	722	0.1	
Ali-Abad	51	1.9	
Gonabad	763	0.3	
Minodasht	69	0	
Kalaleh	75	2.6	0.034

## Discussion

In the present study, we have reported the seroepidemiology of HTLV-I infection in a representative sample of individuals from different cities of the province of Golestan. The overall prevalence of HTLV-I infection in our study is 0.3% (95% CI: 0.06-0.53%) lower than the previous study with 4.4% in Thalassemia patients in Gorgan (14). This is almost in line of many other studies reported from different investigations in the USA 0.6% (15), Brazil 0.3%, China 0.06% (16), Argentina 0.5% (17), Italy 0.6% (18), and Kuwait 0.02% (19) as well as 0.013 in Boshehr (20) and 0.5% in Sistan-Balutchestan, the provinces of Iran, in donated blood (21). In contrast with the above reports, there are similar data indicating higher prevalence in some regions of Iran specially in Mashhad (11- 13), in the east of our region as well as countries such as Japan (6), Ecuador (22), and Karaeeb region (23). In the last 30 years, a lot of studies on the geographic distribution of the HTLV-I have been conducted. Interpretation of the data from the international prevalence studies should be done based on the population selection criteria and diagnostic strategies performed in the research protocol because of their interference with the final result. Mainly, the serological screening of healthy blood donors was the basis for the estimation of the global prevalence of HTLV-I, but it seems to be an underestimation of the seroprevalence in the population (24).

from different investigations in the USA 0.6% (15), Brazil 0.3%, China 0.06% (16), Argentina 0.5% (17), Italy 0.6% (18), and Kuwait 0.02% (19) as well as 0.013 in Boshehr (20) and 0.5% in Sistan-Balutchestan, the provinces of Iran, in donated blood (21). In contrast with the above reports, there are similar data indicating higher prevalence in some regions of Iran specially in Mashhad (11- 13), in the east of our region as well as countries such as Japan (6), Ecuador (22), and Karaeeb region (23). In the last 30 years, a lot of studies on the geographic distribution of the HTLV-I have been conducted. Interpretation of the data from the international prevalence studies should be done based on the population selection criteria and diagnostic strategies performed in the research protocol because of their interference with the final result. Mainly, the serological screening of healthy blood donors was the basis for the estimation of the global prevalence of HTLV-I, but it seems to be an underestimation of the seroprevalence in the population (24).

Comparison of the regional distribution of the carriers in the present study revealed a significant increase of the HTLV-I carriers in Kalaleh area. The observed changes could be considered mainly due to the migration of people from other areas. This interpretation is supported by the observation of ethno-epidemiology studies on HTLV-I carriers who are either born in the endemic areas or the descendants of migrants from those areas. It has been reported that some ethnically defined factors are likely to be associated with HTLV-I diseases among HTLV-I endemic populations (25).

Previous studies have demonstrated that HTLV-I infection increases with age (26-29). Also, there is a linear trend of an increasing age in the general population of Salvador and Brazil, as the endemic areas of HTLV-I infection (30).

In our study, the age distribution of carriers showed presence of HTLV-I carrier in age groups more than 20 years old and the largest number of carriers was observed in this age group. Recent studies showed the number of carriers in the age groups between 0–9 and 50–59 significantly decreased (13). This decline could be explained by changes in the life styles of people such as smaller number of children per family and shorter period of breast feeding. However, exact reasons remain to be elucidated, especially considering the same tendency observed in the study of Brazilian people (30). Taken together, population-based studies on the HTLV-I infection in all age groups suggest that multidisciplinary factors might be involved in virus transmission (13, 31-33).

In conclusion, serological screening of pregnant women, and the blood donors, along with the prevention of mother-to-child infection transmission by stopping breast feeding will greatly reduce the vertical transmission. In addition, there still remain other modalities of HTLV-I infection, such as sexual transmission and possible transuterine infection (34-37). Further comprehensive and detailed epidemiological and molecular study should be considered to provide valuable information based on the risk factors involved in HTLV-I infection and the cost effective vaccine for viable objective of prophylactic intervention in endemic areas.
